# Comparison of ESwab and Wound Fiber Swab Specimen Collection Devices for Use with Xpert SA Nasal Complete Assay

**DOI:** 10.1128/JCM.00449-16

**Published:** 2016-06-24

**Authors:** Eric T. Beck, Blake W. Buchan, Garrett C. Reymann, Nathan A. Ledeboer

**Affiliations:** a Department of Microbiology, Wisconsin Diagnostic Laboratories, Milwaukee, Wisconsin, USA; b Department of Pathology, Medical College of Wisconsin, Milwaukee, Wisconsin, USA; Boston Children's Hospital

## Abstract

Paired nasal swab specimens were collected from patients who were undergoing routine methicillin-resistant Staphylococcus aureus (MRSA) screening prior to elective cardiac or orthopedic procedures. Each patient was swabbed using a traditional wound fiber liquid Stuart swab and an ESwab device, a flocked swab with a modified liquid Amies microbiology transport medium. The two specimens were tested using the Cepheid Xpert SA Nasal Complete assay. Results demonstrated a 95.5% agreement between the ESwab and the FDA-cleared wound fiber swab collection device.

## TEXT

Methicillin-resistant Staphylococcus aureus (MRSA) is an important cause of postsurgical infection. Several recent studies have shown that preoperative screening with nucleic acid amplification tests and subsequent decolonization of MRSA can significantly reduce the rate of postsurgical MRSA infection (1, 2). The Xpert SA Nasal Complete assay (SA Complete) (Cepheid, Sunnyvale, CA) is a molecular test that is approved for screening nasal specimens for the presence of MRSA and methicillin-susceptible S. aureus (MSSA).

As automation of the microbiology laboratory becomes more common, the use of liquid-based microbiology (LBM) specimen transport systems is also increasing. To standardize collection devices across all testing platforms, laboratories must consider validating the use of these devices with molecular tests in addition to culture-based microbiology (3). In our facility, MRSA screening is frequently performed by collecting nasal swab specimens using a liquid Stuart BBL Dual CultureSwab (traditional swab) (BD Diagnostics, Sparks, MD), and testing is performed using the SA Nasal Complete test (one swab is broken off directly into the SA Complete assay elution reagent vial). A previous analytical study indicated that ESwab is a suitable collection device for use with the Xpert MRSA assay (Cepheid) (4). The purpose of the current study was to determine whether an ESwab collection device (ESwab) (Copan Diagnostics, Murrieta, CA) could be used in lieu of a traditional swab for routine MRSA screening from patient nasal swab specimens using the SA Complete assay.

Specimens from patients undergoing MRSA/MSSA screening were collected in parallel with a traditional swab (the FDA-cleared specimen collection device for this assay) and an ESwab. Each swab was placed in both nostrils, with the collection being made in a randomized order (at the collector's discretion). The ESwab collection device consists of a flocked swab, which is used to collect the specimen, and a vial containing 1 ml of modified liquid Amies bacterial transport medium. Following collection, the swab was broken off into the transport medium, which was in turn used as input for the molecular assays. Upon receipt in the laboratory, one of the two traditional swabs from the dual swab collection device was used for testing with the SA Complete assay according to the manufacturer's protocol. ESwab specimens were vortexed, and 200 μl (20% of the ESwab volume) was added to an SA Complete elution reagent vial in lieu of the wound fiber liquid Stuart swab; the remainder of the procedure was carried out according to the manufacturer's product insert (5).

Following testing, the performance of the ESwab was determined by assessing positive and negative percent agreements using the results from the traditional swab as the gold standard. The Xpert MRSA test (performed on the remaining traditional swab from the dual swab collection device) was used as the discriminator for any differences observed in the MRSA result of the SA Complete assay (MSSA discrepant analysis was not performed). The MRSA test detects the same target that the SA Complete assay uses to identify MRSA strains but does not have S. aureus-specific primers/probes. McNemar's test was performed to determine whether the results obtained with the two collection devices were significantly different (two-tailed *P* value < 0.05) (6, 7). In addition to positive and negative percent agreements, the average threshold cycle (*C_T_*) values (amplification cycle number where fluorescence crosses a defined threshold to become positive) for each of the four targets in the assay (specimen processing control [SPC], S. aureus protein A gene [SPA], *mecA*, Staphylococcal cassette chromosome [SCC]) were compared to determine if either collection device had a significantly lower *C_T_* value (i.e., more sensitive). The average *C_T_* values were compared using a two-tailed student's *t* test and were considered significant if the *t* test returned a *P* value of <0.05.

A total of 223 paired specimens were obtained for the study. Seventeen specimens had invalid results that could not be resolved with traditional swabs. Five specimens were invalid with ESwab devices. One specimen was invalid with both swabs. Two specimens were excluded due to inaccurately labeled containers. The final analysis contained 198 specimens that could be compared directly using traditional and ESwab collection devices in the SA Complete assay.

The SA Complete assay provides two separate qualitative results, one for the presence of S. aureus (positive if the SPA target is detected) and one for the presence of methicillin resistance (positive if the SPA, *mecA*, and SCC targets are detected). Specimens that were collected using the ESwab showed a 93.7% and 96.3% positive and negative percent agreement, respectively, with traditional swabs for the detection of S. aureus (Table 1), which is not statistically significant (*P* = 1.0). ESwab showed an 85.7% positive percent agreement and a 100% negative percent agreement for detection of methicillin resistance (Table 2). Discrepant analysis of the MRSA result was performed using the Xpert MRSA assay with the remaining traditional swab, which was taken to be the true result. Following discrepant analysis, the positive and negative percent agreement for methicillin resistance resolved to 92.3% and 100%, respectively (Table 2), which was not statistically significant (*P* = 0.5). A significant limitation of this study was the limited number of positive MRSA specimens (*n* = 13). Future studies of this nature should focus on populations with an increased prevalence of MRSA.

**TABLE 1:** Percent agreement of S. aureus target with traditional and ESwab collection devices in the SA Complete assay^*a*^

Specimen type/result	Traditional swab			Total no.
	No. S. aureus positive	No. S. aureus negative	No. S. aureus invalid	
ESwab				
No. S. aureus positive	59	5	1	65
No. S. aureus negative	4	130	16	150
No. S. aureus invalid	1	4	1	6
Total no.	64	139	18	221

a Positive percent agreement = 59/63 = 93.7% (95% confidence interval [CI], 83.7% to 98.0%). Negative percent agreement = 130/135 = 96.3% (95% CI, 91.1% to 98.6%). Total percent agreement = 189/198 = 95.5%.

**TABLE 2:** Percent agreement of methicillin resistance target with traditional and ESwab collection devices in the SA Complete assay^*a*^

Specimen type/result	Traditional swab			Total no.
	No. *mecA* positive	No. *mecA* negative	No. *mecA* invalid	
ESwab				
No. *mecA* positive	12	0	1	13
No. *mecA* negative	2^*b*^	184	16	202
No. *mecA* invalid	0	5	1	6
Total no.	14	189	18	221

a Positive percent agreement = 12/14 = 85.7% (95% CI, 56.2% to 97.5%). Negative percent agreement = 184/184 = 100% (95% CI, 97.5% to 100.0%). Total percent agreement = 196/198 = 99.0%.

b One specimen was negative for S. aureus and *mecA* with the ESwab specimen and negative for the presence of *mecA* with the Xpert MRSA assay. This represents a false positive with the traditional swab, and the true positive percent agreement is likely 12/13 = 92.3% (95% CI, 62.1% to 99.6%).

In addition to the qualitative results, the *C_T_* values for all of the positive staphylococcal targets (*spa*, *mecA*, and SCC) were recorded. The average *C_T_* values for each target were compared between the two collection devices, and no significant differences in the *C_T_* values were observed despite the fact that only 20% of the ESwab specimens were used as input for the molecular test, while the entire traditional swab specimen is consumed by the assay. The internal process control (SPC) consists of Bacillus globigii spores that are present as a dried cake in each test cartridge to ensure that lysis and thermal cycling conditions are sufficient to release and amplify S. aureus DNA if it is present in the specimen. Because the SPC comes in the test cartridge, the amount present in the assay should be the same for the two collection devices despite the fact that only 20% of the ESwab specimen is loaded into the test. Interestingly, the average *C_T_* value for the SPC was significantly lower (i.e., more sensitive) for the ESwab collection device specimens (*P* = 1.42 × 10^−7^) (Table 3). A more detailed investigation is required to determine the exact nature of this observation. However, some possibilities include a more efficient amplification process with ESwabs, a more complete rehydration of the dried cake and better extraction of the SPC due to an increased volume of liquid being added to the test cartridge, or a decrease in the amount of inhibitors present in the specimen, as only 20% of the ESwab specimen goes into the test.

**TABLE 3:** Comparison of *C_T_* values obtained with traditional and ESwab collection devices for each of the four analytes in the SA Complete assay

Target	No. of specimens^*a*^	Traditional swab		ESwab		*t* test *P* value^*b*^
		Avg *C_T_*	SD of *C_T_*	Avg *C_T_*	SD of *C_T_*	
SPC (internal control)	147	34.64	1.90	33.64	1.20	1.42 × 10^−7^
S. aureus protein A	60	25.43	4.91	25.40	5.10	0.98
*mecA* gene	140	29.93	4.20	29.73	4.55	0.71
SCC cassette	13	25.86	4.90	26.12	6.41	0.91

a These values only included specimens that had an actual *C_T_* value with both the traditional and ESwab collection devices (the SPC does not need to amplify in specimens that are positive for S. aureus, *mecA*, or the SCC cassette.

b Significant at a value of <0.05.

In this study, the ESwab device had 66% fewer invalid results than traditional swabs (6 versus 18, respectively) in the SA Complete assay with no significant differences in sensitivity and specificity. This study suggests that the ESwab collection device is at least equivalent to traditional wound fiber swabs for sample collection and analysis using the Cepheid Xpert SA Nasal Complete assay.
